# Artificial Anus in the Left Lumbar Region

**Published:** 1844-10-19

**Authors:** 


### ARTIFICIAL ANUS IN THE LEFT LUMBAR REGION.

M. Amussat narrated to the academy the following case. Mrs. B., aged fifty-three, of a strong constitution, mother of several children, had never laboured under any serious affection, until the following symptoms manifested themselves:—Obstinate constipation gradually increasing, colics, at first rare, but becoming more and more frequent and intense. From the 12th of last June, there was complete obstruction to the passage of fœcal matter or even gases, although the most powerful purgatives were used. The abdomen became distended, the seat of violent pains, and vomiting set in. Such was the state of things when M. Amussat was consulted. The finger introduced into the rectum discovered no obstacle, no lesion. On examining per vaginam, the neck of the uterus was found to be slightly lowered, the fundus of the organ resting on the rectum, as in retroversion. All the means resorted to having proved vain, it was determined to make an artificial anus, and the left lumbar region was fixed upon, as it was thought that the sigmoid flexure of the colon was, most probably, the seat of the lesion. This diagnosis was founded on the following reasons:—The great frequency of organic lesions of that portion of the descending colon; the absence of fœcal vomiting, which generally exists when an organic affection occupies the small intestines or the superior portion of the large; the impossibility, on the part of the patient, to retain more than a pint of injected fluid, which showed that the obstacle was not situated high up.

The patient was placed on a bed, the abdomen resting on pillows. The crista of the ilium, the margin of the last rib, and the external edge of the sacro-lumbar muscular mass, were marked out with ink. A transverse incision, exposing the aponeurosis of the transversalis, three or four inches in length, was then made. Three small arteries were twisted, and the aponeurosis, having been carefully incised, a small mass of fat escaped, presenting the appearance of an intestinal circumvolution. On cutting through it, a portion of intestine, of a reddish colour, and the external edge of the quadratus lumborum were exposed. A waxed thread having been passed through the intestine, behind the peritoneum, it was raised with a tenaculum and opened. A quantity of gas escaped with a hissing noise, and afterwards fœcal matter appeared. The opening was then enlarged, and the intestine was fixed, by three sutures, to the skin; water was afterwards injected to favour the escape of the fæces. The peritoneum was not opened. The results of the operation were most favourable; not a bad symptom occurred. On the 30th of July, a month after the operation, the state of the patient was most satisfactory. The artificial anus was thoroughly established. During the interval of the evacuations the anus is closed with a wax bougie.

*London Lancet.*

